# Proresolving Mediators LXB4 and RvE1 Regulate Inflammation in Stromal Cells from Patients with Shoulder Tendon Tears

**DOI:** 10.1016/j.ajpath.2019.07.011

**Published:** 2019-11

**Authors:** Stephanie G. Dakin, Romain A. Colas, Kim Wheway, Bridget Watkins, Louise Appleton, Jonathan Rees, Stephen Gwilym, Christopher Little, Jesmond Dalli, Andrew J. Carr

**Affiliations:** ∗ Nuffield Department of Orthopaedics, Rheumatology and Musculoskeletal Sciences, Botnar Research Centre, University of Oxford, Nuffield Orthopaedic Centre, Oxford, United Kingdom; † Lipid Mediator Unit, William Harvey Research Institute, Barts and the London School of Medicine and Dentistry, Queen Mary University of London, London, United Kingdom; ‡ Centre for inflammation and Therapeutic Innovation, Queen Mary University of London, London, United Kingdom

## Abstract

Tendon stromal cells isolated from patients with chronic shoulder rotator cuff tendon tears have dysregulated resolution responses. Current therapies do not address the biological processes concerned with persistent tendon inflammation; therefore, new therapeutic approaches that target tendon stromal cells are required. We examined whether two specialized proresolving mediators (SPMs), lipoxin B4 (LXB4) and resolvin E1 (RvE1), modulate the bioactive lipid mediator profiles of IL-1β–stimulated tendon cells derived from patients with shoulder tendon tears and healthy volunteers. We also examined whether LXB4 or RvE1 treatments moderated the proinflammatory phenotype of tendon tear stromal cells. Incubation of IL-1β–treated patient-derived tendon cells in LXB4 or RvE1 up-regulated concentrations of SPMs. RvE1 treatment of diseased tendon stromal cells increased 15-epi-LXB4 and regulated postaglandin F2α. LXB4 or RvE1 also induced expression of the SPM biosynthetic enzymes 12-lipoxygenase and 15-lipoxygenase. RvE1 treatment up-regulated the proresolving receptor human resolvin E1 compared with vehicle-treated cells. Incubation in LXB4 or RvE1 moderated the proinflammatory phenotype of patient-derived tendon tear cells, regulating markers of tendon inflammation, including podoplanin, CD90, phosphorylated signal transducer and activator of transcription 1, and IL-6. LXB4 and RvE1 counterregulate inflammatory processes in tendon stromal cells, supporting the role of these molecules as potential therapeutics to resolve tendon inflammation.

Diseases of the joint are a considerable global economic burden, accounting for five of the top 15 causes of years lived with disability in well-resourced health care systems.^1^ Shoulder rotator cuff tendon tears are a progressive inflammatory and fibrotic condition, affecting 15% of 60-year–olds and 50% of 80-year–olds.^2^, ^3^ Affected patients experience pain and restricted joint motion, severely limiting activities and disrupting life quality.^4^ Current treatments include physical therapy, nonsteroidal anti-inflammatory drugs, platelet-rich plasma, glucocorticoid injections, and surgery to repair torn tendons. These therapies are frequently ineffective, glucocorticoids are potentially harmful, and tendon tear surgery is associated with high postoperative failure rates.^5^, ^6^, ^7^ Of importance, cyclooxygenase-2 selective nonsteroidal anti-inflammatory drugs dampen protective responses that regulate resolution of inflammation,^8^, ^9^ paradoxically reducing the ability of inflamed tendons to heal. To address this unmet clinical requirement, effective new therapies are required that target the biological mechanisms and cells that drive tendon disease.

Increasing evidence supports the pivotal role of resident stromal cells, including fibroblasts in inflammatory diseases of the joint. Fibroblasts are implicated in the switch from acute to chronic inflammation.^10^ Exposure to an inflammatory milieu induces fibroblasts to undergo phenotypic change whereby these cells exhibit characteristics of an activated state and show capacity for inflammation memory.^11^, ^12^ Cross-talk between fibroblasts with tissue resident macrophages, infiltrating immune cells, and endothelial cells via cytokine and chemokine gradients in inflamed joint tissues further promotes the development of persistent inflammation.^13^, ^14^ We recently identified tendon stromal cells isolated from patients with shoulder tendon tears exhibit a proinflammatory phenotype, highly expressing markers of fibroblast activation and proinflammatory molecules, including IL-6 and signal transducer and activator of transcription (STAT)-1.^12^, ^15^, ^16^ Cells isolated from patients with shoulder tendon tears had dysregulated resolution responses compared with respective cells isolated from the tendons of healthy volunteers.^15^ Specialized proresolving mediators (SPMs), including 15-epi-lipoxin A4 (LXA4) and maresin 1 (MaR1), counterregulate these dysregulated resolution responses and moderate the proinflammatory phenotype of diseased tendon cells.^15^ This study identified that SPMs, including lipoxin B4 (LXB4) and E series resolvins, were differentially regulated in cultures of tendon stromal cells isolated from patients with shoulder tendon tears compared with cells from the tendons of healthy volunteers. Furthermore, low levels of the resolvin E1 (RvE1) were identified in these incubations.^15^ The aim of the current study was to identify new therapeutic approaches to target pathogenic stromal cells and promote resolution of inflammation in cells isolated from patients with shoulder tendon tears. We investigated whether proresolving mediators, including LXB4 and RvE1, target tendon stromal cells (CD45^−^CD34^−^ cells), which comprise most cell types in tendons and are implicated in the pathobiology of tendon disease.^12^ We provide evidence that these SPMs regulate the proinflammatory phenotype and promote resolution responses in patient-derived tendon stromal cells.

## Materials and Methods

### Study Approval

Tendon tissues were collected from patients under research ethics from the Oxford Musculoskeletal Biobank (09/H0606/11). Full informed consent according to the Declaration of Helsinki was obtained from all patients.

### Collection of Patient Tendon Tissues

Patients with rotator cuff shoulder tendon tears were recruited from orthopedic referral clinics. Patients in whom nonoperative treatment failed, including a course of physical therapy, and who had experienced pain for a minimum of 3 months were studied. The presence of a supraspinatus tendon tear was identified by ultrasonography scan. Patients completed the Oxford Shoulder Score, a validated and widely used clinical outcome measure, which was scored from 0 (severe pathologic condition) to 48 (normal function). Supraspinatus tendon tears were collected at the time of surgical debridement of the edges of the torn tendons from 15 male and female patients aged 46 to 75 years (means ± SD, 57 ± 16.3 years of age). All patients were symptomatic and had small to medium supraspinatus tendon tears (≤1 to ≤3 cm in anteroposterior length). Exclusion criteria for all patients in this study included previous shoulder surgery, other shoulder pathologic condition, and inflammatory arthritis. Patients with diabetes and those receiving systemic anticoagulant therapy were also excluded from the study. Samples of healthy volunteer hamstring tendons were collected from 10 male and female patients undergoing surgical reconstruction of their anterior cruciate ligament. All healthy volunteer patients were 20 to 45 years of age (means ± SD, 27.2 ± 10 years of age).

### Isolation of Tendon-Derived Stromal Cells from Healthy and Diseased Tendons

Tendon-derived stromal cells were isolated from the tendons of patients and healthy volunteers using previously published protocols.^12^, ^16^ For experiments, cells were incubated in Dulbecco’s modified Eagle’s medium (DMEM) F12 media (Gibco, Leicestershire, UK) that contained 1% heat inactivated human serum (Sigma, Welwyn Garden City, UK) and 1% penicillin-streptomycin. Passage 1 to 3 cells were used for all experiments. Tendon stromal cells were previously characterized as CD45^neg^ and CD34^neg^ cells that exhibited fibroblast morphologic features.^12^

### Cytokine Treatment of Tendon-Derived Stromal Cells

To induce an inflammatory milieu, tendon stromal cells were stimulated with IL-1β because it is known to induce expression of NF-κB target genes that are highly expressed in shoulder tendon disease.^16^ The bioactive lipid mediator (LM) profiles in tendon-derived stromal cells isolated from the tendons of healthy volunteers and patients with shoulder tendon tears were investigated in the presence of 10 ng mL^−1^ of IL-1β (Sigma) in medium (DMEM F12 phenol red–free medium, Gibco), containing 1% heat-inactivated human serum (Sigma) and 1% penicillin-streptomycin. Vehicle controls were stimulated with IL-1β and an equivalent dilution of ethanol, which is the solvent for LXB4 and RvE1. After cytokine/vehicle treatment, cells were incubated at 37°C and 5% carbon dioxide for 24 hours until experimental harvest of the media and lysate for bioactive LM profiling.

### Modulating Bioactive LM Profiles of IL-1β–Stimulated Tendon-Derived Stromal Cells with LXB4 and RvE1

Tendon stromal cells were isolated from healthy volunteers or patients with tendon tears (*n* = 5 each) and seeded at a density of 60,000 cells per well. Once cells were 80% confluent, they were preincubated with 10 nmol/L LXB4 (Cayman Chemical, Ann Arbor, MI) or 10 nmol/L RvE1 (Cayman Chemical) for 24 hours in DMEM F12 phenol red–free medium (Gibco) that contained 1% heat inactivated human serum (Sigma) and 1% penicillin-streptomycin. Cells were stimulated with IL-1β (10 ng mL^−1^) in the presence of media that contained LXB4, RvE1, or vehicle control as previously described.^15^ Parallel experiments were performed and cell lysates harvested to investigate whether incubating cells in these SPMs moderated the expression of markers of the proinflammatory phenotype of diseased tendon stromal cells and potentiated expression of SPM synthetic enzymes and receptors mediating resolution of inflammation. The concentration and integrity of mediators used for these incubations were validated using UV spectrophotometry and liquid chromatography with tandem mass spectrometry in accordance with published criteria.^17^ Bioactive LM profiling of media and lysate samples from IL-1β–stimulated tendon cells was performed using a previously described method.^15^ Calibration curves were obtained for each using authentic compound mixtures and deuterium labeled LM at 0.78, 1.56, 3.12, 6.25, 12.5, 25, 50, 100, and 200 pg. Linear calibration curves were obtained for each LM, which gave *r*^2^ values of 0.98 to 0.99.

### Immunocytochemistry for LXB4- and RvE1-Treated Tendon Stromal Cells

**Supplemental Figure S1:**
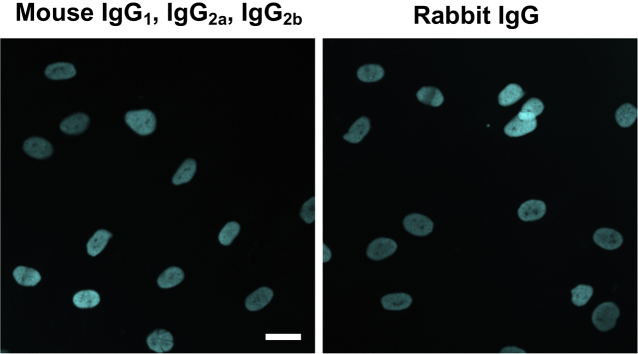
Isotype control staining of patient-derived tendon stromal cells. Representative confocal immunofluorescence images showing merged images of stromal cells isolated from patients with shoulder tendon tears, stained with isotype control antibodies for mouse IgG1, IgG2a, IgG2b, and rabbit IgG fractions. Cyan represents POPO-1 nuclear counterstain. Scale bar = 20 μm.

Tendon stromal cells isolated from patients and healthy volunteers were grown in chamber slides and stimulated with IL-1β in the presence of LXB4, RvE1, or vehicle for 24 hours as described above. Cells were fixed in ice cold methanol for 5 minutes and washed with phosphate-buffered saline. Immunofluorescence staining protocols and image acquisition are adapted from a previously published protocol.^15^ Tendon stromal cells isolated from healthy volunteers and patients with tendon tears (*n* = 3 each) were incubated with the following primary antibodies: anti–lipoxin A4 receptor (ALX) (ab26316), anti–lipoxygenase (ALOX) 15 (ab119774), anti-ALOX12 (, ab211506), anti–human resolvin E1 (ERV1) (ab167097), anti–leukotriene B4 receptor (BLT1) (ab18886), anti–STAT-1 (phosphoY701) (ab29045), anti-podoplanin (PDPN) (ab10288), and anti–IL-6 (ab9324) (Abcam, Cambridge, UK) in phosphate-buffered saline that contained 5% goat serum in saponin for 3 hours at room temperature. For negative controls, the primary antibody was substituted for universal isotype control antibodies: cocktail of mouse IgG1, IgG2a, IgG2b, IgG3, and IgM (Dako, Ely, UK) and rabbit immunoglobulin fraction of serum from nonimmunized rabbits, solid phase absorbed. Isotype control staining is shown in Supplemental Figure S1. Immunofluorescence images were acquired on a Zeiss LSM 710 confocal microscope using a previously published protocol.^15^

### Expression of Proinflammatory and Proresolving Genes in Tendon Stromal Cells Incubated in LXB4 or RvE1

Tendon-derived stromal cells from healthy volunteers or patients with shoulder tendon tears (*n* = 6 each) were seeded at a density of 20,000 cells per well in a 24-well plate. Cells were allowed to reach confluence before preincubation with LXB4 or RvE1 and subsequent stimulation with 10 ng mL^−1^ of IL-1β. Nontreated cells (vehicle only, containing 0.1% endotoxin-free bovine serum albumin; Sigma) served as controls for each experiment. After treatment, cells were incubated at 37°C in 5% carbon dioxide until harvest of the cell lysate for mRNA after 24 hours. RNA isolation, cDNA synthesis, and quantitative PCR were performed using previously published protocols.^16^ Prevalidated primer assays (*ALOX15*, *ERV1*, *IL6*, *PDPN*, *CD90*, β-actin, and *GAPDH*) from Qiagen (Manchester, UK) were used for quantitative PCR. Results were calculated using the ΔΔCt method using reference genes for human β-actin and *GAPDH*. Results were consistent using these reference genes, and data are shown normalized to β-actin.

### Quantification of IL-6 in Tissue Culture Media

IL-6 is an important cytokine implicated in inflammation and is abundantly released by tendon stromal cells isolated from patients with shoulder tendon tears after stimulation with IL-1β.^15^ IL-6 in tissue culture supernatants was measured using enzyme-linked immunosorbent assay reagents (BD Biosciences, Franklin Lakes, NJ) using incubations isolated from five donors. Minimum detectable IL-6 concentration for this assay was 2.2 pg mL^−1^. Optical density was read on a spectrophotometric enzyme-linked immunosorbent assay plate reader (FLUOstar Omega, BMG Labtech, Ortenberg, Germany) and analyzed using MARS data analysis software version 5.10R2 (BMG Labtech, Ortenberg, Germany).

### Phospho-Signaling in LXB4- and RvE1-Treated Tendon Stromal Cells

A human phosphokinase array kit (ARY003B; R&D Systems) was used to investigate the effects of incubating IL-1β–stimulated patient-derived tendon cells in LXB4 or RvE1 on protein kinase signaling pathways (*n* = 3 donors). Experimental protocols were performed according to the manufacturer's instructions on protein lysates harvested after 24 hours of incubation in LXB4 or RvE1. Images were captured using a chemiluminescence documentation system (UVITEC, Cambridge, UK), and densitometry analysis of proteins of interest was performed using ImageJ software version 1.47v (ImageJ bundled with Java 1.8.0_172, NIH, Bethesda, MD; *https://imagej.nih.gov/ij*).

### Statistical Analysis

Statistical analyses were performed using GraphPad Prism version 7.0 (GraphPad Software Inc., San Diego, CA). Normality was tested using the Shapiro-Wilk normality test. Analysis of bioactive LM profiles from tendon cells derived from patients and healthy volunteers was performed using multivariate statistical analysis, orthogonal partial least-squares discriminant analysis using SIMCA software version 14.1 (Umetrics, Umea, Sweden) following unit variance scaling of LM amounts. Partial least-squares discriminant analysis is based on a linear multivariate model that identifies variables that contribute to class separation of observations (cell incubations) on the basis of their variables (LM levels). During LM classification, observations were projected onto their respective class model. The score plot illustrates the systematic clusters among the observations (closer plots presenting higher similarity in the data matrix). Loading plot interpretation identified the variables with the best discriminatory power (variable importance in projection >1) that were associated with tight clusters for LM profiles obtained from incubations with cells from healthy volunteers or patients with tendinopathy. For levels of proresolving mediators and inflammation initiating eicosanoids, data are shown as summed with SEM, where *n* is the biological replicate. Unpaired *t*-tests were used to test for differences in LM levels between tendon cells derived from healthy volunteers and patients with shoulder tendon tears. Pairwise *U*-tests were used to determine differences in expression of proinflammatory and proresolving genes and IL-6 protein in IL-1β–treated tendon stromal cells in the presence or absence of LXB4, RvE1, or respective vehicle. *P* < 0.05 was considered statistically significant.

## Results

### LXB4 and RvE1 Treatments Induce SPM Release from Tendon-Derived Stromal Cells

**Figure 1:**
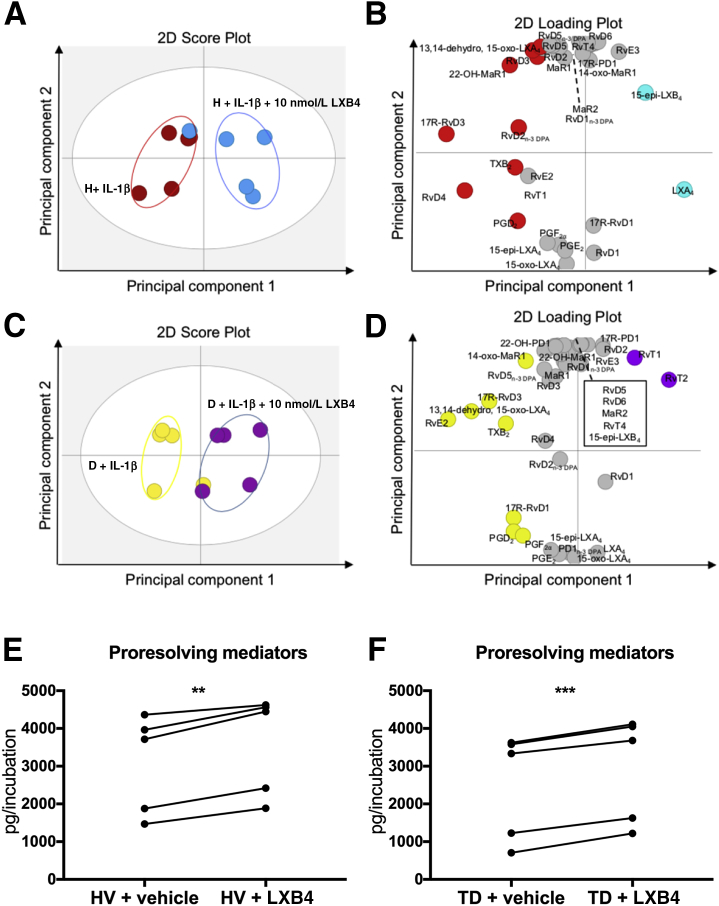
Lipoxin B4 (LXB4) up-regulates specialized proresolving mediator (SPM) concentrations in IL-1β–stimulated tendon stromal cells. Tendon stromal cells were derived from healthy volunteers (H) and patients with shoulder tendon tears [diseased (D)]. Cells were incubated with 10 nmol/L LXB4 or vehicle for 24 hours at 37°C then with 10 ng mL^−1^ of IL-1β for 24 hours. Lipid mediators (LMs) were identified and quantified using LM profiling. Two-dimensional (2D) score plot (**A**) and corresponding SD loading plot (**B**) of LM-SPMs from human tendon–derived stromal cell incubations isolated from the H group incubated with IL-1β and 10 nmol/L LXB4 or vehicle only. 2D score plot (**C**) and corresponding 2D loading plot (**D**) of LM-SPMs from human tendon–derived stromal cell incubations isolated from the D group incubated with IL-1β and 10 nmol/L LXB4 or vehicle only. Cumulative concentrations of proresolving mediators [docosahexaenoic acid–derived resolvin D (RvD), protectins (PD), maresin (MaR), n-3 docosapentaenoic acid (DPA) –derived RvD_n-3 DPA_, PD_n-3 DPA_, MaR_n-3 DPA_, EPA-derived RvE, and arachadonic acid–derived LX] in IL-1β–stimulated tendon stromal cell incubations in the presence of 10 nmol/L LXB4 or vehicle for 24 hours. LXB4 up-regulated concentrations of SPMs compared with vehicle only in H (**E**) and D (**F**) tendon stromal cells. Results are representative of *n* = 5 donors per group. **P < 0.01, ***P < 0.001. TD, tendon disease.

**Figure 2:**
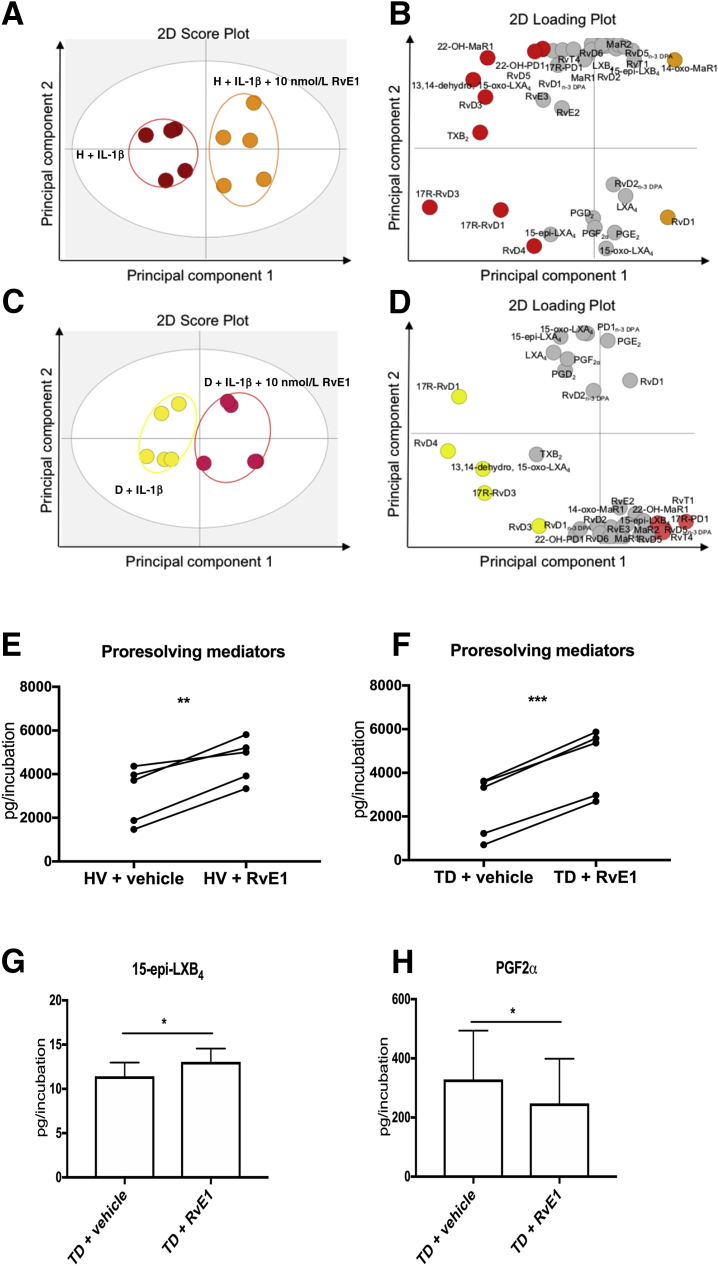
Resolvin E1 (RvE1) increases specialized proresolving mediator (SPM) levels in IL-1β–stimulated tendon stromal cells. Tendon stromal cells were derived from healthy volunteers (H) and patients with shoulder tendon tears [diseased (D)]. Cells were incubated with 10 nmol/L RvE1 or vehicle for 24 hours at 37°C and then with 10 ng mL^−1^ of IL-1β for 24 hours. Lipid mediators (LMs) were identified and quantified using LM profiling. Two-dimensional (2D) score plot (**A**) and corresponding 2D loading plot (**B**) of LM-SPM from human tendon–derived stromal cell incubations isolated from healthy volunteers (H) incubated with IL-1β and 10 nmol/L RvE1 or vehicle only. 2D score plot (**C**) and corresponding 2D loading plot (**D**) of LM-SPM from human tendon–derived stromal cell incubations isolated from the D group incubated with IL-1β and 10 nmol/L RvE1 or vehicle only. Cumulative concentrations of proresolving mediators [docosahexaenoic acid–derived resolvin D (RvD), protectins (PD), maresin (MaR), n-3 docosapentaenoic acid (DPA) –derived RvD_n-3 DPA_, PD_n-3 DPA_, MaR_n-3 DPA_, EPA-derived RvE, and arachadonic acid–derived LX] in IL-1β–stimulated tendon stromal cell incubations in the presence of 10 nmol/L RvE1 or vehicle for 24 hours. RvE1 up-regulated concentrations of SPMs compared with vehicle only in the H (**E**) and D (**F**) tendon stromal cells. Differentially regulated lipid mediators in IL-1β–stimulated diseased tendon stromal cell incubations in the presence of 10 nmol/L RvE1 or vehicle for 24 hours of RvE1 treatment up-regulated 15-epi-LXB4 (**G**) and reduced prostaglandin F2α (PGF2α) levels (**H**) compared with vehicle controls. Data are expressed as means ± SEM. *n* = 5 donors per group. *P < 0.05, **P < 0.01, and ***P < 0.001. TD, tendon disease.

**Supplemental Figure S2:**
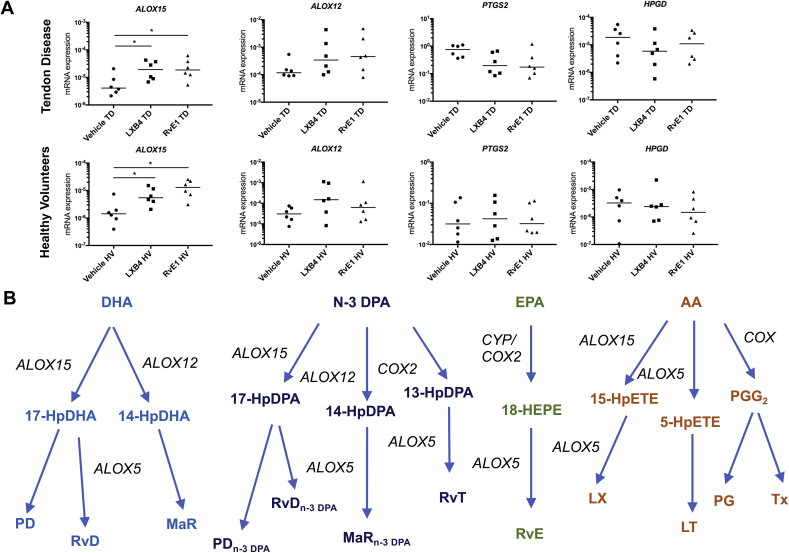
Biosynthesis of specialized proresolving mediators in tendon stromal cells. **A:** mRNA expression of enzymes implicated in the biosynthesis of specialized proresolving mediator (SPMs; *ALOX15*, *ALOX12*), inflammation-initiating eicosanoids (*PTGS2*), and the degradation of SPMs and inflammation-initiating eicosanoids (*HPGD*) in tendon stromal cells from patients with shoulder tendon tears and healthy volunteers (HVs). Incubation in lipoxin B4 (LXB4) significantly induces *ALOX15* mRNA in both tendon disease (TD) and HV cells compared with respective vehicle controls. Incubation in resolvin E1 (RvE1) significantly induces *ALOX15* mRNA in both TD and HV cells compared with respective vehicle controls. Gene expression is normalized to β-actin. **B:** Summary of lipid mediator biosynthetic pathways. **Bars** indicate median values. *P < 0.05 versus respective vehicle control. PD, protectin; RvT, 13-series resolvin; MaR, maresin; RvE, E-series resolvins; LX, lipoxins; LT, leukotrienes; PG, prostaglandins; Tx, thromboxane; ALOX, lipoxygenase; COX, cyclooxygenase; CYP, cytochrome P450.

Tendon stromal cells isolated from patients with shoulder tendon tears have dysregulated resolution responses compared with cells isolated from healthy volunteer tendons.^15^ This study found that SPMs, including LXB4 and E series resolvins, were differentially regulated in healthy and diseased tendon stromal cells and identified low levels of RvE1 in these incubations. To gain further insights into whether these SPMs counterregulate tendon inflammation, we investigated whether LXB4 and RvE1 modulated the bioactive LM profiles of IL-1β–stimulated tendon stromal cells isolated from the tendons of patients with shoulder tendon tears and healthy volunteers. Multivariate analysis identified differences in bioactive LM profiles between IL-1β–stimulated tendon cells isolated from healthy volunteer donors or patients with tendon tears in the presence of 10 nmol/L LXB4 compared with vehicle-only incubations, demonstrated by the distinct clustering of the LM profiles (Figure 1, A–D, Supplemental Figure S2). The molecules profiled, together with the concentrations of the individual LMs identified, are listed in Supplemental Table S1. In these incubations, LXB4 up-regulated concentrations of SPM in IL-1β–stimulated tendon cells from healthy volunteers (*P* < 0.001) (Figure 1E) and patients with tendon tears (*P* < 0.001) (Figure 1F). Multivariate analysis also identified distinct clustering of the LM profiles between IL-1β–stimulated tendon cells isolated from healthy volunteer donors and patients with tendon tears incubated in the presence of 10 nmol/L RvE1 compared with vehicle only (Figure 2, A–D). The molecules profiled, together with the concentrations of the individual LMs identified, are listed in Supplemental Table S1. In these incubations, RvE1 up-regulated concentrations of SPMs in healthy volunteer tendon cells (*P* < 0.01) (Figure 2E) and patients with tendon tears (*P* < 0.001) (Figure 2F). In tendon tear incubations, RvE1 up-regulated the concentrations of specific SPMs, including 15-epi-LXB4 (*P* < 0.05) (Figure 2G) and decreased levels of the proinflammatory eicosanoid prostaglandin F2α (PGF2α) (*P* < 0.05) (Figure 2H). Incubation of healthy tendon stromal cells in LXB4 or RvE1 did not significantly change levels of 15-epi-LXB4 or PGF2α compared with vehicle-only incubations.

### LXB4 and RvE1 Up-Regulate the Expression of SPM Biosynthetic Enzymes and Proresolving Receptors in Tendon-Derived Stromal Cells

**Figure 3:**
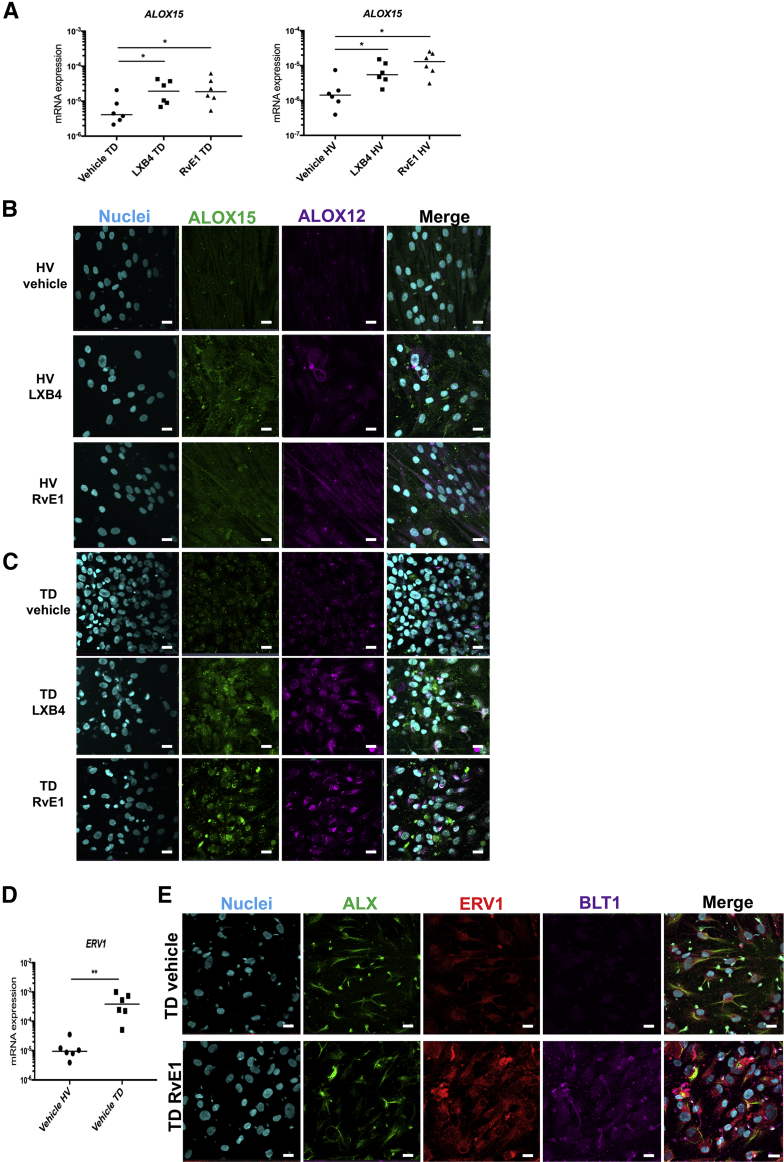
Lipoxin B4 (LXB4) and resolvin E1 (RvE1) induce specialized proresolving mediator (SPM) biosynthetic enzymes and regulate the proresolving receptor for chemerin and resolvin E1 (ChemR23)/human resolvin E1 (ERV1) in tendon stromal cells. Tendon stromal cells were derived from patients with shoulder tendon tears (TD; *n* = 6) or healthy volunteers (HV; *n* = 6). Cells were incubated with 10 nmol/L LXB4, 10 nmol/L RvE1, or vehicle for 24 hours at 37°C then with 10 ng mL^−1^ of IL-1β for 24 hours. **A:** Incubation in LXB4 significantly induces *ALOX15* mRNA in both TD and HV cells compared with respective vehicle controls. Incubation in RvE1 significantly induces *ALOX15* mRNA in both TD and HV cells compared with respective vehicle controls. Gene expression is normalized to β-actin. Representative images of immunocytochemistry for the SPM biosynthetic enzymes 15-lipoxygenase (ALOX15) (green) and 12-lipoxygenase (ALOX12) (violet) in IL-1β–stimulated HV (**B**) and TD tendon stromal cells (**C**) incubated in 10 nmol/L LXB4, 10 nmol/L RvE1, or vehicle control for 24 hours. Cyan represents POPO-1 nuclear counterstain. **D:** IL-1β–stimulated TD cells show increased *ERV1* mRNA expression compared with respective HV cells. Gene expression is normalized to β-actin. **E:** Representative images of immunocytochemistry for proresolving receptors lipoxin A4 receptor (ALX) (green), human resolvin E1 (ERV1) (red), and leukotriene B4 receptor (BLT1) (violet) in IL-1β–stimulated diseased tendon stromal cells incubated in 10 nmol/L LXB4, 10 nmol/L RvE1, or vehicle control for 24 hours. Cyan represents POPO-1 nuclear counterstain. Bars indicate median values. *n* = 3 donors. Scale bars = 20 μm (**B**, **C**, and **E**). *P < 0.05, **P < 0.01.

**Supplemental Figure S3:**
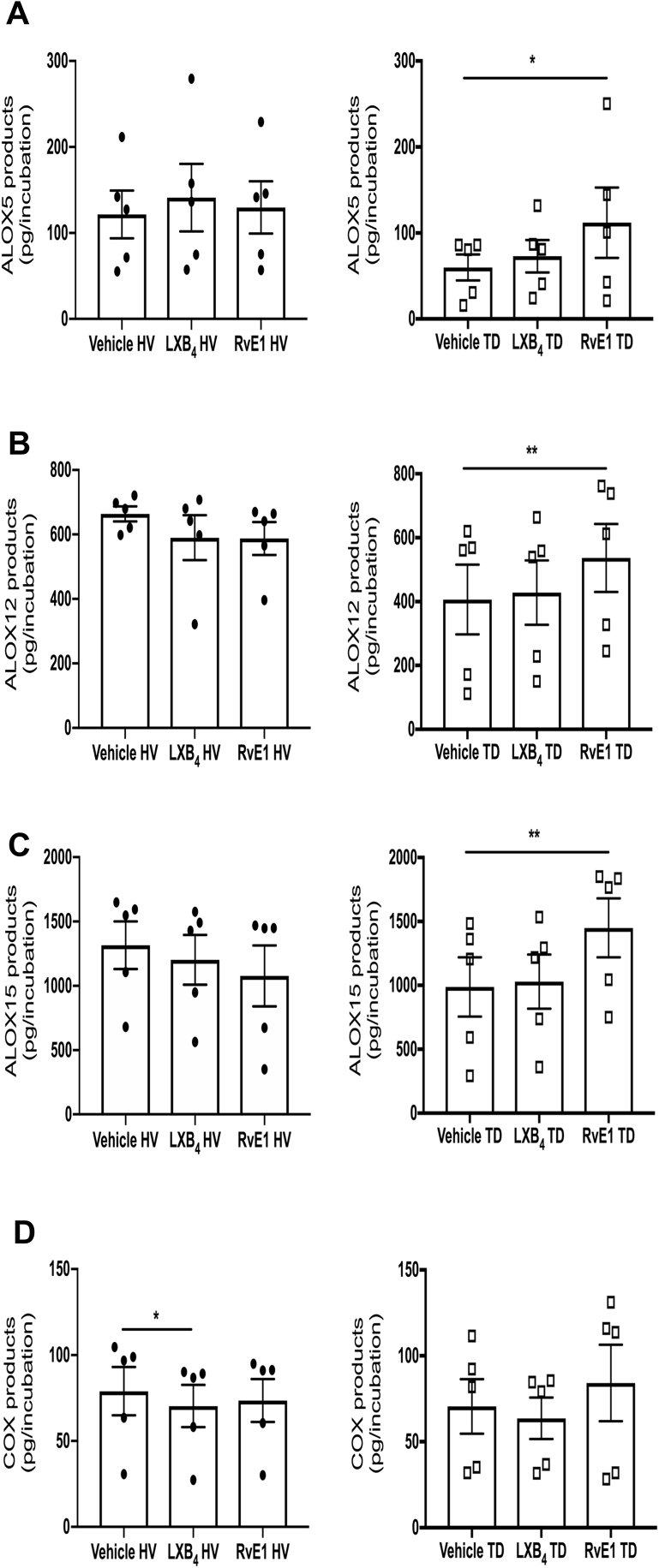
Differential regulation of specialized proresolving mediator (SPM) biosynthetic enzyme activity by resolvin E1 (RvE1) and lipoxin B4 (LXB4) in patient-derived and healthy (H) volunteer tendon stromal cells. Tendon stromal cells were derived from the H group (*n* = 5 donors) and patients with shoulder tendon tears or the diseased (D) group (*n* = 5 donors). Cells were incubated with 10 nmol/L RvE1, 10 nmol/L LXB4, or vehicle for 24 hours at 37°C then with 10 ng mL^−1^ of IL-1β for 24 hours. Monohydroxy fatty acids–produced SPM biosynthetic enzymes were identified and quantified using liquid chromatography with tandem mass spectrometry–based lipid mediator profiling. Cumulative concentrations of the 5-lipoxygenase (ALOX5) products 7-hydroxydocosahexaenoic acid, 7-hydroxydocosapentaenoic acid, 5-hydroxyeicosapentaenoic acid, and 5-hydroxyeicosatetraenoic acid (**A**); the 12-lipoxygenase (ALOX12) products 14-hydroxydocosahexaenoic acid, 14-hydroxydocosapentaenoic acid, 12-hydroxyeicosapentaenoic acid, and 12-hydroxyeicosatetraenoic acid (**B**); the 15-lipoxygenase (ALOX15) products 17-hydroxydocosahexaenoic acid, 17-hydroxydocosapentaenoic acid, 15-hydroxyeicosapentaenoic acid, and 15-hydroxyeicosatetraenoic acid (**C**); and cyclooxygenase (COX) products 17-hydroxydocosahexaenoic acid and 17-hydroxydocosapentaenoic acid (**D**). Data are expressed as means ± SEM. *P < 0.05, **P < 0.01 versus respective vehicle control (Friedman's test followed by Dunn's *post hoc* test).

The mechanisms by which LXB4 and RvE1 up-regulated SPMs were next investigated. Incubation of IL-1β–stimulated tendon cells isolated from patients with tendon tears in LXB4 or RvE1 induced *ALOX15* mRNA expression relative to respective vehicle controls (*P* < 0.05) (Figure 3A). The same treatment of healthy volunteer tendon cells also up-regulated *ALOX15* mRNA expression relative to respective vehicle controls (*P* < 0.05) (Figure 3A). Immunostaining found that these treatments also increased expression of ALOX12 and ALOX15 proteins implicated in the biosynthesis of SPMs (Figure 3, B and C). Induction of these biosynthetic enzymes was profound in cells isolated from patients with tendon tears compared with healthy volunteer donors (Figure 3, B and C). To gain further insights into the regulation of SPM pathways, the activity of the SPM biosynthetic enzymes was also assessed, measuring the concentrations of their monohydroxy products in the tendon stromal cell incubations. In incubations with cells from healthy volunteers, the expression of ALOX5 products 7-hydroxydocosahexaenoic acid, 7-hydroxydocosapentaenoic acid, 5-hydroxyeicosapentaenoic acid, and 5-hydroxyeicosatetraenoic acid was not changed by the addition of either LXB_4_ or RvE1. Concentrations of these pathway markers were up-regulated in patient-derived tendon cell incubations in the presence of RvE1 (Supplemental Figure S3A). Concentrations of the ALOX12 products 14-hydroxydocosahexaenoic acid, 14-hydroxydocosapentaenoic acid, 12-hydroxyeicosapentaenoic acid, and 12-hydroxyeicosatetraenoic acid were decreased by LXB4 or RvE1 in cells from heathy volunteers, whereas RvE1 increased the concentrations of these molecules in cells from patients with tendon tears (Supplemental Figure S3B). Similar results were observed when assessing the concentrations of the ALOX15 and cyclooxygenase products (Supplemental Figure S3, C and D).

Whether incubation of tendon tear cells in RvE1 moderated expression of receptors to which RvE1 is known to bind was also investigated. In the presence of vehicle only, IL-1β–stimulated tendon tear cells increased *CHEMR23/ERV1* mRNA compared with respective healthy tendon cells (*P* < 0.01) (Figure 3D). Indeed, RvE1 treatment up-regulated the expression of receptor for chemerin and resolvin E1 (ChemR23)/ERV1 and BLT1 receptors in IL-1β–stimulated tendon tear cells compared with respective vehicle controls (Figure 3E).

### LXB4 and RvE1 Moderate the Proinflammatory Phenotype of Tendon Stromal Cells

**Figure 4:**
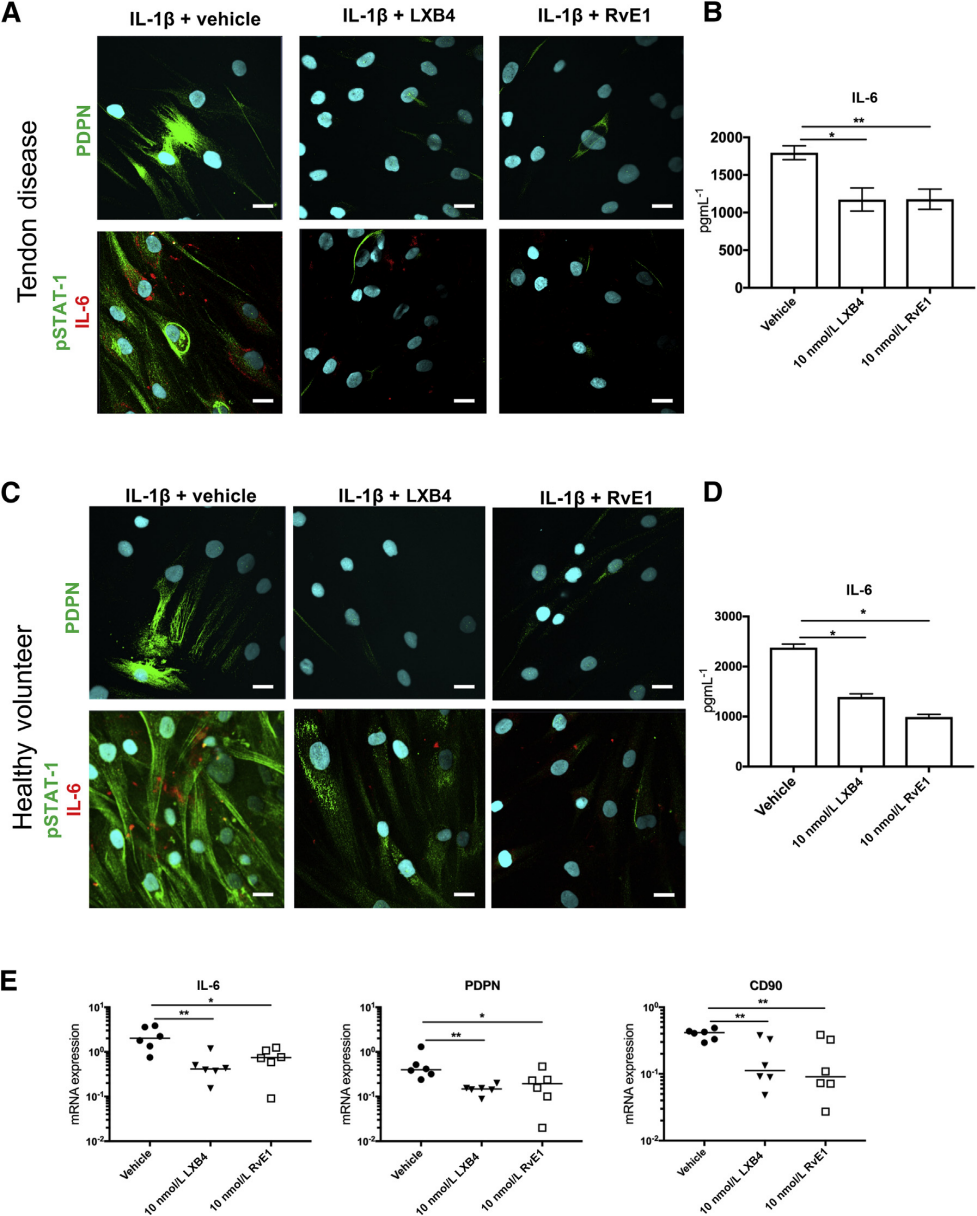
Lipoxin B4 (LXB4) and resolvin E1 (RvE1) moderate the proinflammatory phenotype of tendon stromal cells. Tendon stromal cells were derived from patients with shoulder tendon tears (TD) or healthy volunteers (HV). **A:** Representative images of immunocytochemistry for established markers of tendon inflammation, including podoplanin (PDPN) (green), phosphorylated signal transducer and activator of transcription (pSTAT)-1 (green), and IL-6 (red) in IL-1β–stimulated diseased tendon stromal cells incubated in 10 nmol/L LXB4, 10 nmol/L RvE1, or vehicle control for 24 hours. Cyan represents POPO-1 nuclear counterstain. **B:** Enzyme-linked immunosorbent assay of IL-6 protein secretion from IL-1β–stimulated diseased tendon cells incubated in the presence and absence of 10 nmol/L LXB4 or 10 nmol/L RvE1. LXB4 or RvE1 reduces IL-6 levels compared with vehicle only. **C:** Representative images of immunocytochemistry for PDPN, pSTAT-1, and IL-6 in IL-1β–stimulated healthy stromal cells incubated in 10 nmol/L LXB4, 10 nmol/L RvE1, or vehicle control for 24 hours. Cyan represents POPO-1 nuclear counterstain. **D:** LXB4 or RvE1 reduces IL-6 levels in HV incubations compared with vehicle only. **E:** mRNA expression of markers of tendon inflammation, including IL-6, and the fibroblast activation markers PDPN and CD90 in IL-1β–stimulated TD cells incubated in 10 nmol/L LXB4, 10 nmol/L RvE1, or vehicle control for 24 hours. Incubation in LXB4 or RvE1 reduces *IL6*, *PDPN*, and *CD90* mRNA expression compared with vehicle controls. Gene expression is normalized to β-actin. Data are expressed as means ± SEM. **Bars** indicate median values. *n* = 3 donors (**A** and **C**); *n* = 5 donors (**B** and **D**); *n* = 6 donors (**E**). Scale bars = 20 μm (**A** and **C**). *P < 0.05, **P < 0.01.

**Figure 5:**
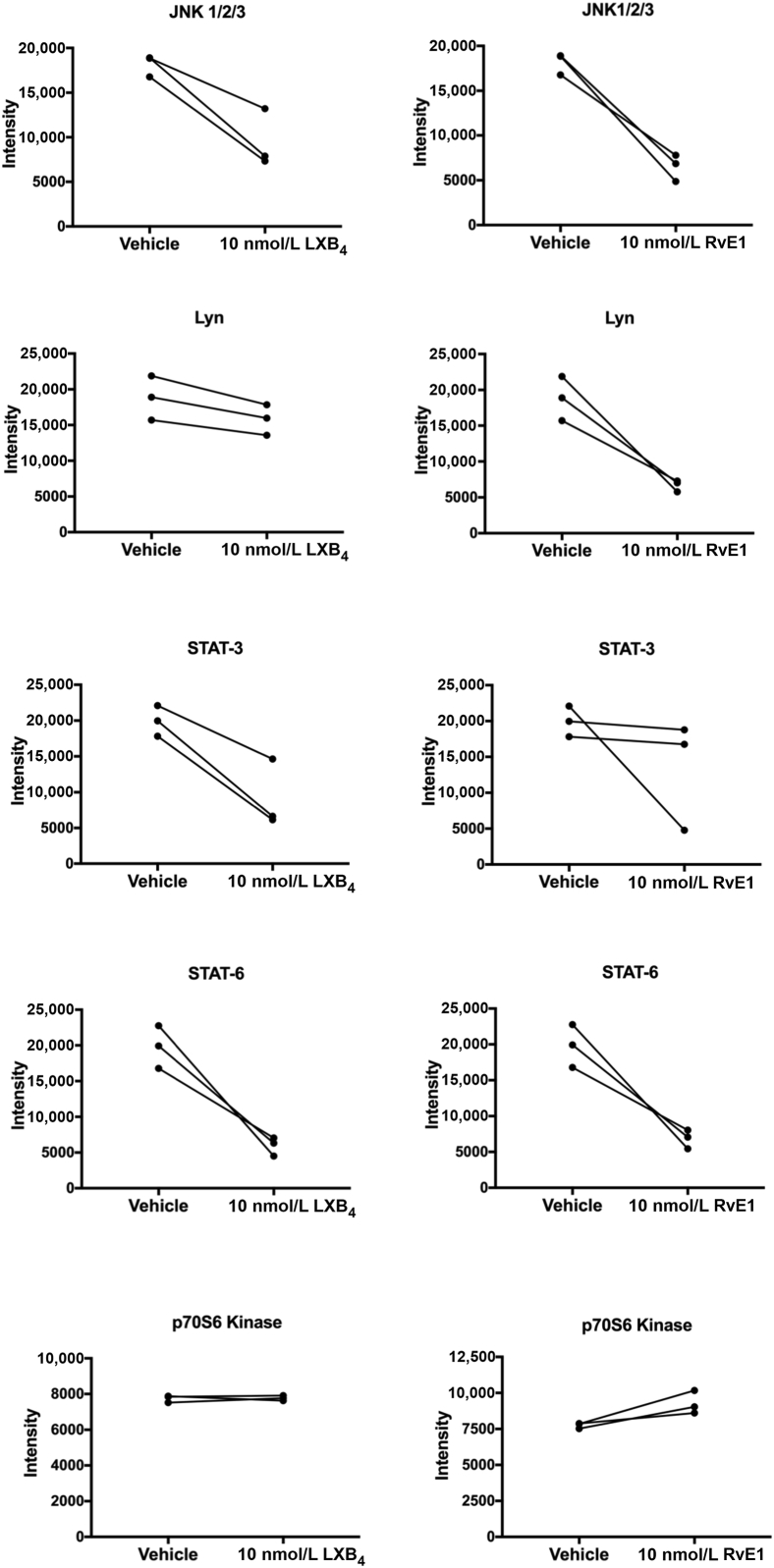
Effects of Lipoxin B4 (LXB4) and resolvin E1 (RvE1) on protein phosphokinase signaling in diseased tendon stromal cells. Densitometric analysis was acquired using ImageJ software version 1.47 to identify the effects of incubating IL-1β–treated tendon disease (TD) cells in 10 nmol/L LXB4 or RvE1 on protein phosphokinase signaling pathways JNK1/2/3, Lyn, signal transducer and activator of transcription (STAT)-3, STAT6, and p70s6 kinase. Data are expressed as means ± SEM. *n* = 3 donors per group relative to respective vehicle control treated cells.

**Supplemental Figure S4:**
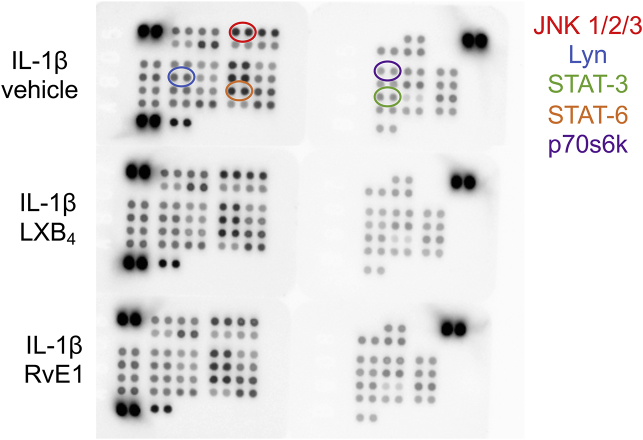
Effects of lipoxin B4 (LXB4) and resolvin E1 (RvE1) on protein kinase expression in diseased tendon stromal cells. The image showing human phosphokinase array (ARY003B; R&D Systems, Minneapolis, MN) was used to detect phosphorylated proteins in lysates of IL-1β–stimulated diseased tendon stromal cells in the presence of 10 nmol/L LXB4, RvE1, or vehicle for 24 hours. Data are shown from an 8-minute exposure. Image is representative of data from incubated cells derived from one donor. Quantitative analysis of data from three donors is shown in Figure 5. Human phosphokinase array coordinates are listed in the R&D Systems product insert Proteome Profiler Array, Human Phospho-Kinase Array Kit. Minneapolis, MN. Colored circles show phosphorylated proteins of interest shown in Figure 5.

It was next assessed whether LXB4 and RvE1 also regulate known markers of tendon inflammation in tendon cells isolated from patients with tendon tears and healthy volunteers. Incubation of IL-1β–stimulated diseased cells in LXB4 or RvE1 for 24 hours reduced fibroblast activation marker PDPN, pSTAT-1, and IL-6 compared with respective vehicle controls (Figure 4A). Measurement of IL-6 levels in supernatant from IL-1β–stimulated diseased cells indicated that incubation in LXB4 or RvE1 reduced IL-6 levels compared with vehicle only (*P* < 0.05 and *P* < 0.01, respectively) (Figure 4B). The same treatment of IL-1β–stimulated healthy tendon cells also reduced PDPN, pSTAT-1, and IL-6 compared with respective vehicle controls (Figure 4C). In the incubations of healthy volunteers, LXB4 or RvE1 treatment also reduced IL-6 levels (*P* < 0.05) (Figure 4D). It was next determined whether LXB4 or RvE1 treatment moderated expression of proinflammatory genes and signaling pathways in IL-1β–stimulated tendon tear cells. Incubation of these cells in LXB4 reduced *IL6*, *PDPN*, and *CD90* mRNA expression compared with vehicle controls (*P* < 0.01, *P* < 0.01, and *P* < 0.05 respectively) (Figure 4E). RvE1 treatment of IL-1β–stimulated tendon tear cells also reduced *IL6*, *PDPN*, and *CD90* mRNA compared with vehicle controls (*P* < 0.05) (Figure 4E). LXB4 or RvE1 treatments did not significantly modulate expression of phosphokinase signaling pathways, including JNK1/2/3 (phosphorylation sites T183/Y185, T221/Y223), Lyn (Y397), STAT-3 (Y705), STAT-6 (Y641), and p70S6 (T389), compared with respective vehicle control treated cells (Figure 5). A representative human phosphokinase array used to detect phosphorylated proteins in lysates of IL-1β–stimulated diseased tendon stromal cells in the presence of 10 nmol/L LXB4, RvE1, or vehicle is shown in Supplemental Figure S4.

## Discussion

Resident stromal cells, including fibroblasts, are increasingly recognized as important cell types that drive chronic inflammatory joint disease.^10^, ^18^, ^19^ After exposure to an inflammatory milieu, tendon and synovial fibroblasts adopt a proinflammatory phenotype and exhibit inflammation memory.^11^, ^12^, ^20^ Distinct fibroblast subtypes that mediate joint inflammation and tissue damage have been characterized in rheumatoid synovium.^21^ Recent advances in the knowledge of how resident stromal cells behave under inflammatory conditions of the joint have prompted further investigation of the resolution responses of these cells. Given that stromal fibroblasts comprise most cell types of musculoskeletal soft tissues, improved understanding of how these cells respond to an inflammatory milieu is required to inform the development of therapeutic strategies that target these cells. It was recently identified that tendon stromal cells isolated from patients with tendon tears had increased levels of SPMs and inflammation initiating eicosanoids compared with cells isolated from the tendons of healthy volunteers, reminiscent of a dysregulated resolution response characteristic of chronic inflammation.^15^ It was also identified that incubation of IL-1β–stimulated tendon stromal cells in 15-epi-LXA4 or MaR1 regulated proinflammatory eicosanoids and potentiated the further release of SPMs.^15^ These treatments moderated the proinflammatory phenotype of IL-1β–stimulated diseased tendon stromal cells, dampening expression of PDPN, pSTAT-1, and IL-6. SPMs, including LXB4 and E series resolvins, are differentially regulated between IL-1β–stimulated tendon cells isolated from patients with shoulder tendon tears compared with cells from the tendons of healthy volunteers, identifying low levels of RvE1 in these incubations.^15^ In the current study, it was therefore investigated whether LXB4 or RvE1 modulated the bioactive LM profiles of IL-1β–stimulated tendon cells derived from these patient cohorts. Tendon stromal cells were stimulated with IL-1β because it is known to induce expression of NF-κB target genes highly expressed in human tendon disease,^16^, ^20^ simulating an inflammatory environment. During inflammasome activation, phospholipase A2 is known to regulate eicosanoid class switching.^22^ In tendon stromal cell incubations, treatment with LXB4 or RvE1 up-regulated SPM concentrations. RvE1 treatment specifically increased 15-epi-LXB4 and regulated PGF2α in incubations of IL-1β–stimulated diseased tendon cells. The mechanism of action underpinning these observations was examined next. Incubating in LXB4 or RvE1 induced expression of SPM biosynthetic enzymes ALOX12 and ALOX15 in healthy and diseased tendon stromal cells. Notably, expression of these SPM biosynthetic enzymes was increased in diseased compared with healthy tendon stromal cells. Incubation of tendon stromal cells in 15-epi-LXA4 or MaR1 induces ALOX15 expression.^15^ The findings from the current study support these observations, suggesting a common mechanism whereby proresolving mediators activate feed forward cascades, leading to the up-regulation of other SPMs via induction of ALOX biosynthetic enzymes. We next investigated whether incubating tendon cells in LXB4 or RvE1 influenced expression of proresolving receptors. The receptors to which LXB4 binds have not yet been identified, although RvE1 is known to activate ERV1 and competitively inhibit BLT1.^23^, ^24^ In the absence of SPM treatment, *ERV1* mRNA expression was increased in diseased compared with healthy IL-1β–stimulated tendon stromal cells, suggesting that a proinflammatory phenotype favors increased expression of this proresolving receptor. Incubation of LXB4 or RvE1 did not induce ALX expression, although RvE1 treatment further up-regulated ERV1 and BLT1 expression on tendon stromal cells compared with vehicle-treated cells. Collectively, these findings suggest a positive feedback loop, whereby RvE1 treatment up-regulates ERV1 and BLT1 receptor expression. This process may occur as a direct consequence of RvE1 treatment or via RvE1-induced up-regulation of SPM.

Incubation in LXB4 or RvE1 moderated the proinflammatory phenotype of patient-derived tendon tear cells, regulating known markers of tendon inflammation, including PDPN, CD90, pSTAT-1, and IL-6. CD90 is known to be highly expressed by rheumatoid synovial fibroblasts that exhibit a proinflammatory and invasive phenotype.^21^ This fibroblast activation marker is highly expressed by diseased but not healthy tendons and likely represents a subset of tendon cells that exhibit a proinflammatory phenotype.^20^ CD90 is therefore a conserved marker of fibroblast activation that is up-regulated during inflammatory disease of soft tissues of the joint.^13^ Persistent fibroblast activation may be implicated in the development of chronic tendon inflammation and increased likelihood of recurrent injury.^12^ Proresolving mediators may therefore possess therapeutic utility to moderate the proinflammatory phenotype of tendon stromal cells via attenuating expression of fibroblast activation markers. In support of this, other SPMs, including 15-epi-LXA4, also moderated PDPN expression in IL-1β–stimulated diseased tendon cells,^15^ suggesting that this property is common in different families of SPMs, including the lipoxins and resolvins.

Therapies that promote resolution of inflammation are an important future therapeutic strategy to address pathogenic stroma in chronic inflammatory joint disease. The proresolving mediator resolvin D3 regulates leukocyte infiltration and proinflammatory eicosanoids in murine inflammatory arthritis.^25^ The 17R epimer of RvD1 also attenuated arthritis severity, paw edema, and leukocyte infiltration in acute murine inflammatory arthritis.^26^ The current study suggests that LXB4 and RvE1 regulate expression of tendon proinflammatory molecules, including podoplanin, CD90, pSTAT-1, and IL-6, and dampen phosphokinases, including JNK1/2/3, Lyn, STAT-3, and STAT-6. LXB4 and RvE1 treatments also up-regulated SPMs in IL-1β–stimulated healthy and diseased tendon cells. The concentrations of SPMs identified in the present experiments are within their bioactive ranges,^27^, ^28^ and the magnitudes of the responses of diseased cells are likely to be biologically relevant. Although the incubation of tendon cells up-regulated SPM production in both healthy and diseased cells, the LMs up-regulated by each of the SPMs in cells from the different groups are characteristic as indicated by the orthogonal partial least-squares discriminant analysis. Indeed, incubating tendon cells from healthy volunteers with LXB4 up-regulated 15-epi-LXB4 and LXA4, whereas cells from patients with shoulder tendon tears incubated with LXB4 up-regulated RvT1 and RvT2. Similar findings were also made when healthy volunteer– and patient-derived cells were incubated with RvE1. Together these findings suggest that although diseased tendon cells display an altered resolution response, this can be rectified by the addition of resolution agonists, such as LXB4 or RvE1, promoting the termination of tendon inflammation. We therefore propose that SPMs, including LXB4 and RvE1, are potential new therapeutics to target pathogenic stromal cells and potentiate resolution of chronic tendon inflammation.
