# Supplementary material for: Proresolving Mediators LXB4 and RvE1 Regulate Inflammation in Stromal Cells from Patients with Shoulder Tendon Tears
Source: Am J Pathol. 2019 Nov;189(11):2258–68. doi: 10.1016/j.ajpath.2019.07.011 (PMC6876268; doi:10.1016/j.ajpath.2019.07.011)
Supplement: Supplemental Table S1 [file mmc1.docx]

**Supplemental Table S1. LM-SPM profiles of IL-1β–stimulated patient tendon stromal cells in the presence of LXB_4_ or RvE1.**

| **Tendon stromal cells lipid mediator levels**  **pg/incubation** | **Healthy IL1β** | | | **Healthy IL1β+LXB_4_** | | | **Healthy IL1β+RvE1** | | | **Disease IL1β** | | | **Disease IL1β +LXB_4_** | | | **Disease IL1β +RvE1** | | |
| --- | --- | --- | --- | --- | --- | --- | --- | --- | --- | --- | --- | --- | --- | --- | --- | --- | --- | --- |
| **DHA bioactive metabolome** | Mean | ± | SEM | Mean | ± | SEM | Mean | ± | SEM | Mean | ± | SEM | Mean | ± | SEM | Mean | ± | SEM |
| RvD1 | 3.69 | ± | 0.27 | 3.72 | ± | 0.36 | 4.01 | ± | 0.61 | 3.27 | ± | 0.11 | 3.41 | ± | 0.29 | 3.97 | ± | 0.63 |
| RvD2 | 3.60 | ± | 0.60 | 3.37 | ± | 0.71 | 3.65 | ± | 0.68 | 3.15 | ± | 0.43 | 3.02 | ± | 0.57 | 3.30 | ± | 0.62 |
| RvD3 | 5.72 | ± | 0.67 | 5.15 | ± | 0.92 | 5.08 | ± | 0.83 | 4.98 | ± | 0.89 | 5.21 | ± | 0.60 | 4.34 | ± | 0.76* |
| RvD4 | 9.61 | ± | 0.89 | 8.05 | ± | 0.77 | 8.63 | ± | 1.25 | 8.04 | ± | 0.61 | 7.77 | ± | 1.00 | 6.18 | ± | 0.73* |
| RvD5 | 49.22 | ± | 3.45 | 45.75 | ± | 3.51* | 46.03 | ± | 4.77 | 39.56 | ± | 6.76 | 40.38 | ± | 8.11 | 46.65 | ± | 11.32 |
| RvD6 | 17.05 | ± | 2.21 | 15.82 | ± | 2.17 | 17.42 | ± | 2.56 | 12.66 | ± | 3.33 | 12.38 | ± | 3.53 | 14.09 | ± | 4.54 |
| 17R-RvD1 | 1.80 | ± | 0.09 | 1.75 | ± | 0.35 | 1.58 | ± | 0.35 | 2.02 | ± | 0.30 | 1.46 | ± | 0.30 | 1.23 | ± | 0.20* |
| 17R-RvD3 | 7.77 | ± | 0.40 | 6.79 | ± | 0.54 | 6.29 | ± | 0.63* | 6.56 | ± | 1.07 | 6.03 | ± | 0.92 | 5.14 |  | 0.58 |
| PD1 | 0.00 | ± | 0.00 | 0.00 | ± | 0.00 | 0.00 | ± | 0.00 | 0.00 | ± | 0.00 | 0.00 | ± | 0.00 | 0.00 | ± | 0.00 |
| 10S,17SdiHDHA | 194.78 | ± | 52.68 | 186.02 | ± | 51.43 | 208.14 | ± | 64.86 | 155.75 | ± | 64.89 | 171.52 | ± | 71.24 | 209.10 | ± | 89.95 |
| 22OHPD1 | 18.49 | ± | 6.26 | 14.68 | ± | 5.01* | 16.65 | ± | 5.91* | 16.55 | ± | 5.83 | 15.16 | ± | 5.38 | 14.91 | ± | 5.23 |
| 17R-PD1 | 3.64 | ± | 1.31 | 3.17 | ± | 1.16 | 3.45 | ± | 1.54 | 2.86 | ± | 1.14 | 3.60 | ± | 1.45 | 3.93 | ± | 1.81 |
| Maresin1 | 4.36 | ± | 1.44 | 4.61 | ± | 2.17 | 4.44 | ± | 1.73 | 4.18 | ± | 2.07 | 2.80 | ± | 1.87 | 4.31 | ± | 2.06 |
| Maresin2 | 7.31 | ± | 1.23 | 7.24 | ± | 1.05 | 7.67 | ± | 1.49 | 6.46 | ± | 1.36 | 6.28 | ± | 1.33 | 7.42 | ± | 2.20 |
| 22-OH-MaR1 | 14.93 | ± | 1.14 | 12.91 | ± | 1.94 | 12.55 | ± | 1.83* | 14.51 | ± | 2.19 | 13.74 | ± | 2.79 | 15.83 | ± | 1.53 |
| 14-oxo-MaR1 | 0.12 | ± | 0.05 | 0.22 | ± | 0.12 | 0.26 | ± | 0.12* | 0.15 | ± | 0.08 | 0.08 | ± | 0.04 | 0.13 | ± | 0.07 |
| 7S,14S diHDHA | 57.82 | ± | 12.55 | 58.35 | ± | 16.73 | 63.33 | ± | 14.94 | 45.00 | ± | 13.70 | 54.77 | ± | 20.03 | 65.74 | ± | 23.07* |
| 4,14-diHDHA | 55.78 | ± | 14.89 | 50.98 | ± | 13.88* | 51.92 | ± | 13.59 | 37.43 | ± | 13.38 | 35.82 | ± | 12.94 | 40.80 | ± | 15.85 |
| **n-3 DPA bioactive metabolome** | | | | | | | | | | | | | | | | | | |
| RvT1 | 0.34 | ± | 0.16 | 0.25 | ± | 0.14 | 0.46 | ± | 0.22 | 0.24 | ± | 0.12 | 0.40 | ± | 0.20 | 0.52 | ± | 0.25 |
| RvT2 | 0.00 | ± | 0.00 | 0.07 | ± | 0.08 | 0.06 | ± | 0.06 | 0.07 | ± | 0.08 | 0.26 | ± | 0.12 | 0.04 | ± | 0.05 |
| RvT3 | 0.00 | ± | 0.00 | 0.00 | ± | 0.00 | 0.00 | ± | 0.00 | 0.00 | ± | 0.00 | 0.00 | ± | 0.00 | 0.00 | ± | 0.00 |
| RvT4 | 14.32 | ± | 3.50 | 13.78 | ± | 3.61 | 14.83 | ± | 3.83 | 13.21 | ± | 3.15 | 13.65 | ± | 3.60 | 16.23 | ± | 5.49 |
| RvD1_n3 DPA_ | 7.54 | ± | 1.26 | 7.01 | ± | 0.91 | 7.26 | ± | 0.80 | 6.63 | ± | 1.14 | 7.09 | ± | 0.91 | 6.34 | ± | 1.22 |
| RvD2_n3 DPA_ | 1.54 | ± | 0.47 | 1.28 | ± | 0.42 | 1.87 | ± | 0.35 | 1.74 | ± | 0.73 | 1.30 | ± | 0.47 | 1.55 | ± | 0.33 |
| RvD5_n3 DPA_ | 30.26 | ± | 3.19 | 27.79 | ± | 2.97* | 32.86 | ± | 5.54 | 27.14 | ± | 4.38 | 27.78 | ± | 4.95 | 32.21 | ± | 8.04 |
| PD1_n3 DPA_ | 0.00 | ± | 0.00 | 0.00 | ± | 0.00 | 0.00 | ± | 0.00 | 0.62 | ± | 0.43 | 0.40 | ± | 0.28 | 0.50 | ± | 0.37 |
| 10S,17S-diHDPA | 19.08 | ± | 5.20 | 20.59 | ± | 4.75 | 21.85 | ± | 6.21 | 16.40 | ± | 4.83 | 17.32 | ± | 5.45 | 22.76 | ± | 7.84* |
| MaR1_n3 DPA_ | 0.00 | ± | 0.00 | 0.00 | ± | 0.00 | 0.00 | ± | 0.00 | 0.00 | ± | 0.00 | 0.00 | ± | 0.00 | 0.00 | ± | 0.00 |
| 7S,14S-diHDPA | 53.09 | ± | 10.49 | 50.59 | ± | 8.68 | 59.71 | ± | 15.52 | 41.39 | ± | 12.98 | 41.42 | ± | 14.33 | 53.73 | ± | 20.22 |
| **EPA bioactive metabolome** |  |  |  |  |  |  |  |  |  |  |  |  |  |  |  |  |  |  |
| RvE1 | 0.00 | ± | 0.00 | 0.00 | ± | 0.00 | 1397.39 | ± | 315.54 | 0.00 | ± | 0.00 | 0.00 | ± | 0.00 | 1306.85 | ± | 325.88 |
| RvE2 | 33.49 | ± | 1.82 | 32.39 | ± | 1.80 | 31.46 | ± | 2.60 | 37.50 | ± | 1.88 | 32.74 | ± | 3.15* | 37.98 | ± | 2.17 |
| RvE3 | 3.12 | ± | 0.67 | 3.46 | ± | 0.41 | 3.13 | ± | 0.67 | 2.58 | ± | 0.55 | 2.59 | ± | 0.97 | 3.29 | ± | 1.10 |
| **AA bioactive metabolome** |  |  |  |  |  |  |  |  |  |  |  |  |  |  |  |  |  |  |
| LXA_4_ | 2.02 | ± | 0.16 | 2.39 | ± | 0.30 | 2.15 | ± | 0.22 | 1.68 | ± | 0.26 | 1.75 | ± | 0.20 | 1.42 | ± | 0.19 |
| LXB_4_ | 3.84 | ± | 1.85 | 396.95 | ± | 90.14* | 3.43 | ± | 1.88 | 0.00 | ± | 0.00 | 330.76 | ± | 102.83 | 0.00 | ± | 0.00 |
| 5,15-diHETE | 2332.88 | ± | 550.67 | 2496.44 | ± | 629.07 | 2509.74 | ± | 681.76 | 1860.42 | ± | 589.74 | 1967.32 | ± | 667.77 | 2461.83 | ± | 908.88 |
| 15-epi-LXA_4_ | 56.73 | ± | 7.92 | 50.34 | ± | 6.70* | 49.89 | ± | 9.07* | 54.85 | ± | 9.78 | 51.37 | ± | 10.54 | 46.96 | ± | 7.07* |
| 15-epi-LXB_4_ | 12.52 | ± | 2.10 | 13.37 | ± | 1.08 | 12.82 | ± | 1.80 | 11.42 | ± | 1.74 | 10.90 | ± | 1.87 | 13.05 | ± | 1.68* |
| 13,14-dihydro-15-oxo-LXA_4_ | 28.45 | ± | 4.08 | 23.87 | ± | 4.04* | 22.27 | ± | 3.65* | 29.75 | ± | 3.14 | 25.87 | ± | 3.29 | 25.64 | ± | 1.23 |
| 15-oxo-LXA_4_ | 22.42 | ± | 15.50 | 18.62 | ± | 12.75 | 22.04 | ± | 15.10 | 25.43 | ± | 17.48 | 22.32 | ± | 15.31 | 18.91 | ± | 13.61 |
| LTB_4_ | 0.00 | ± | 0.00 | 0.00 | ± | 0.00 | 0.00 | ± | 0.00 | 0.00 | ± | 0.00 | 0.00 | ± | 0.00 | 0.00 | ± | 0.00 |
| 5,12 diHETE | 333.87 | ± | 61.15 | 324.79 | ± | 57.51 | 332.24 | ± | 58.63 | 261.78 | ± | 76.72 | 260.65 | ± | 77.52 | 277.98 |  | 90.72 |
| 6-trans-LTB_4_ | 133.21 | ± | 12.31 | 124.72 | ± | 14.20 | 126.85 | ± | 12.30 | 134.89 | ± | 29.45 | 131.64 | ± | 28.06 | 139.94 | ± | 32.82 |
| 12-epi-6-trans-LTB_4_ | 164.92 | ± | 28.04 | 146.95 | ± | 25.43 | 149.35 | ± | 23.89 | 152.83 | ± | 42.66 | 150.40 | ± | 40.20 | 164.86 | ± | 49.33 |
| PGD_2_ | 359.42 | ± | 160.23 | 297.71 | ± | 83.52 | 327.95 | ± | 114.56 | 386.28 | ± | 91.64 | 348.86 | ± | 73.43 | 332.98 | ± | 61.52 |
| PGE_2_ | 6475.47 | ± | 4794.74 | 6574.62 | ± | 4996.22 | 6342.04 | ± | 4649.23 | 8854.74 | ± | 3776.51 | 9020.79 | ± | 4111.85 | 9503.78 | ± | 3911.65* |
| PGF_2α_ | 273.17 | ± | 227.86 | 227.45 | ± | 191.61 | 221.54 | ± | 179.65 | 328.78 | ± | 184.29 | 277.58 | ± | 176.02 | 247.95 | ± | 168.56* |
| TXB_2_ | 10.20 | ± | 0.84 | 9.21 | ± | 0.71* | 9.19 | ± | 0.52 | 14.32 | ± | 3.36 | 13.92 | ± | 1.87 | 11.78 | ± | 1.32 |

Tendon stromal cells (60,000 cells per well) were derived from patients with shoulder tendon tears (Diseased n=5 donors) or healthy volunteer hamstring tendons (Healthy n=5 donors) and incubated in the presence of 10nM LXB_4_, 10nM RvE1, or vehicle only in the presence of 10ngmL^-1^ IL1β. Cell incubations were terminated using ice-cold methanol containing internal standards and lipid mediators (LM) were identified and quantified using LM-profiling (see Materials and Methods). Results are expressed as pg/incubation. Mean ± SEM of n = 5 per incubation. **P* ≤ 0.05 comparison between respective vehicle incubated tendon stromal cells. The detection limit was ~ 0.1 pg. 0.0, below limits of detection.
